# Supplementary material for: The structure of the mammalian bornavirus polymerase complex
Source: Nat Commun. 2025 Aug 13;16:7508. doi: 10.1038/s41467-025-62906-4 (PMC12350838; doi:10.1038/s41467-025-62906-4)
Supplement: Supplementary file 1 — Supplementary Information [file 41467_2025_62906_MOESM1_ESM.pdf]

The structure of the mammalian bornavirus polymerase complex

Loïc Carrique[1*], Franziska Günl[2*], Adrian Deng[3], Jonathan M. Grimes[1], Jeremy R. Keown[1,3]

1 Division of Structural Biology, Centre for Human Genetics, University of Oxford, Oxford, United Kingdom
2 Sir William Dunn School of Pathology, University of Oxford, Oxford, United Kingdom
3 School of Life Sciences, University of Warwick, Coventry, United Kingdom

*These authors contributed equally to this work: Loïc Carrique and Franziska Günl

email: jeremy.keown@warwick.ac.uk

Supplementary information. Table 1 and Figures 1-6.

	Sample 1 – RNA free Full-length<br>EMD-51765<br>9H1G	Sample 2 - core<br>EMD-51770<br>9H1Q	Sample 2 – Full-length<br>EMD-51785<br>9H1Y
**Data collection**			
Microscope	Titan Krios G3i (OPIC)		
Voltage (kV)	300		
Detector	Falcon 4 - SelectrisX		
Magnification	130,000		
Movie/micrograph pixel size (Å)	0.932		
Dose rate (e-/px/sec)	6.2		
Movie exposure time (s)	7		
Total dose (e-/Å^2)	50		
Defocus range (um)	1.4 to 2.6		
**EM data processing**			
Number of movies/micrographs	17,665	14,249	
Box size (px)	300		
Particle number (total)	428,267	588,492	
Particle number (used in final map)	158,264	264,568	106,976
Symmetry	C1		
Map resolution (Å, FSC 0.143)	3.07	2.95	3.07
Local resolution range (Å)	2.8 - 30	2.8 - 30	2.8 - 30
Map sharpening B-factor (Å^2)	121	125	111
**Model Building and Validation**			
Initial model used	AlphaFold2	9H1G	9H1G
Model composition			
Non-hydrogen protein atoms	14581	10313	14036
Protein residues	1848	1302	1777
B factors (Å^2) - min/max/mean			
Protein	64.1/361/193	69.3/261/145	77.9/403/188
RMSD from ideal			
Bond length (Å)	0.004	0.004	0.004
Bond angles (°)	0.496	0.533	0.520
Validation			
Molprobity score	1.93	1.78	1.9
Clashscore	9.1	8.26	9.6
FSC (0.5) model-vs-map	3.6	3.3	3.7
CCmodel-vs-map (mask)	0.74	0.77	0.77
Ramachandran plot			
Favored (%)	93.1	95.3	94.3
Allowed (%)	6.9	4.7	5.7
Outliers (%)	0	0	0

Supplementary Table 1. CryoEM collection and refinement parameters

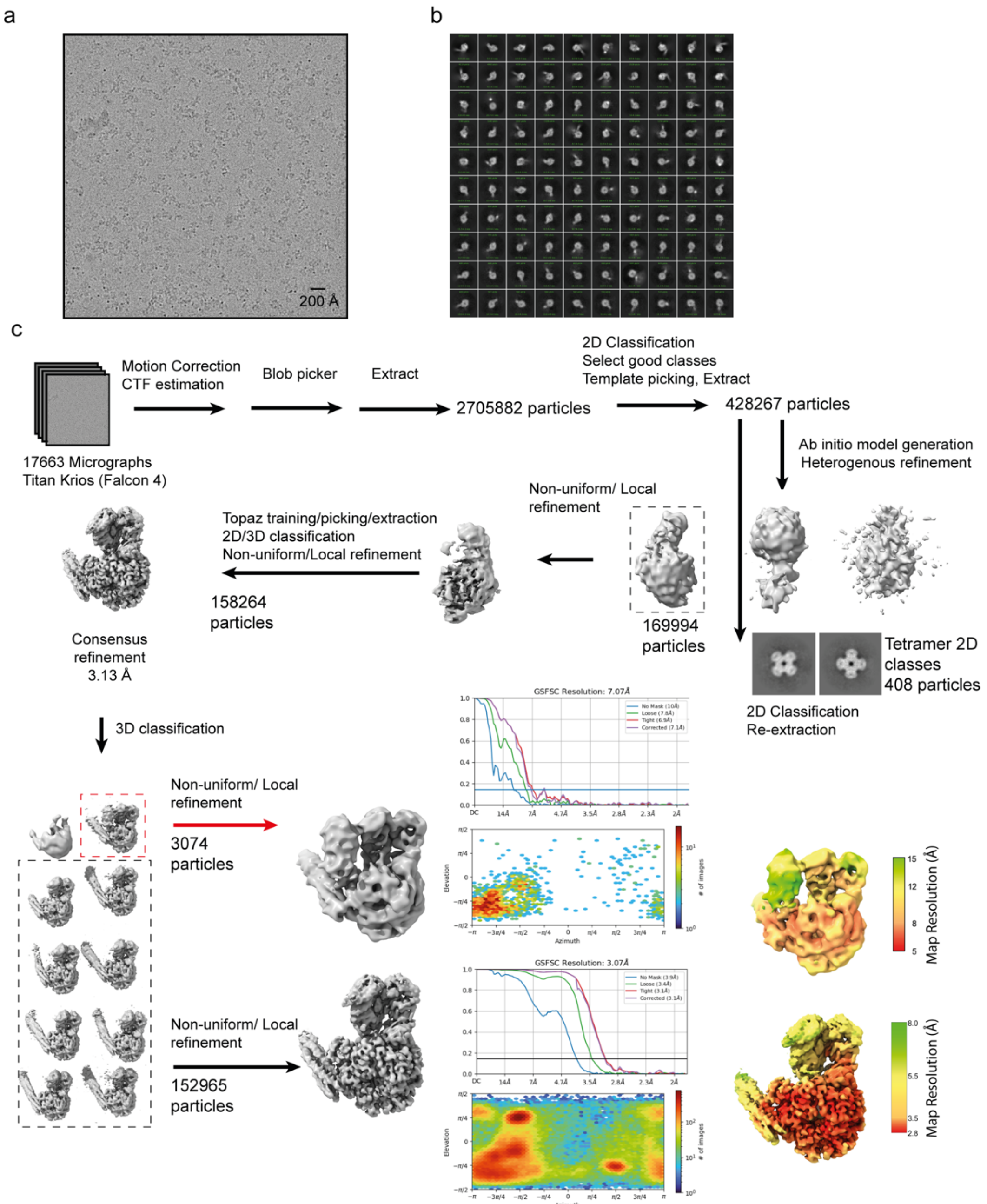

Supplementary figure 1. Sample 1 CryoEM processing workflow. a) representative electron micrograph image. b) 2D classification result showing the diversity of orientations present. c) CryoEM processing scheme.

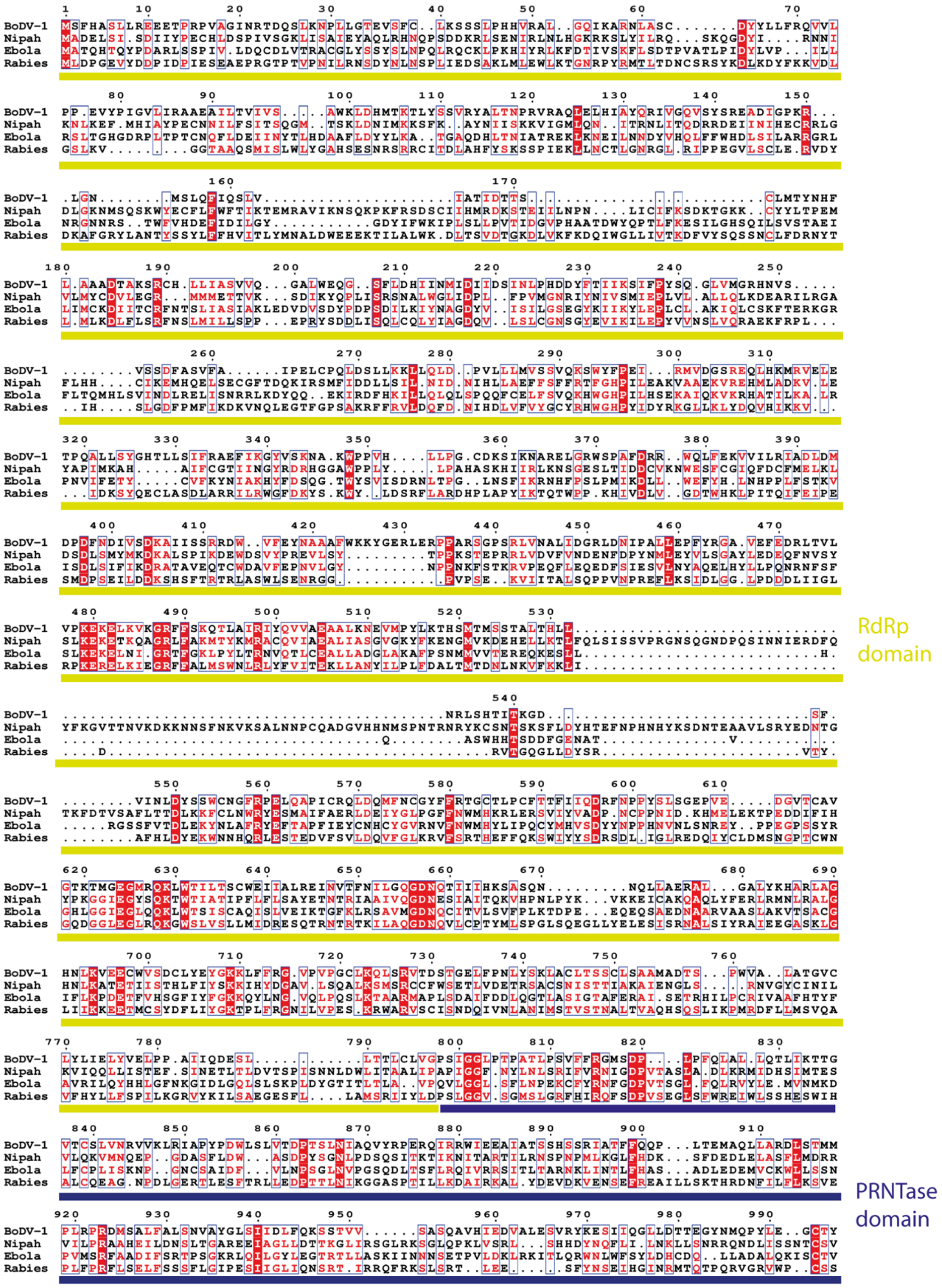
BoDV-1
Nipah
Ebola
Rabies
RdRp domain
PRNTase domain

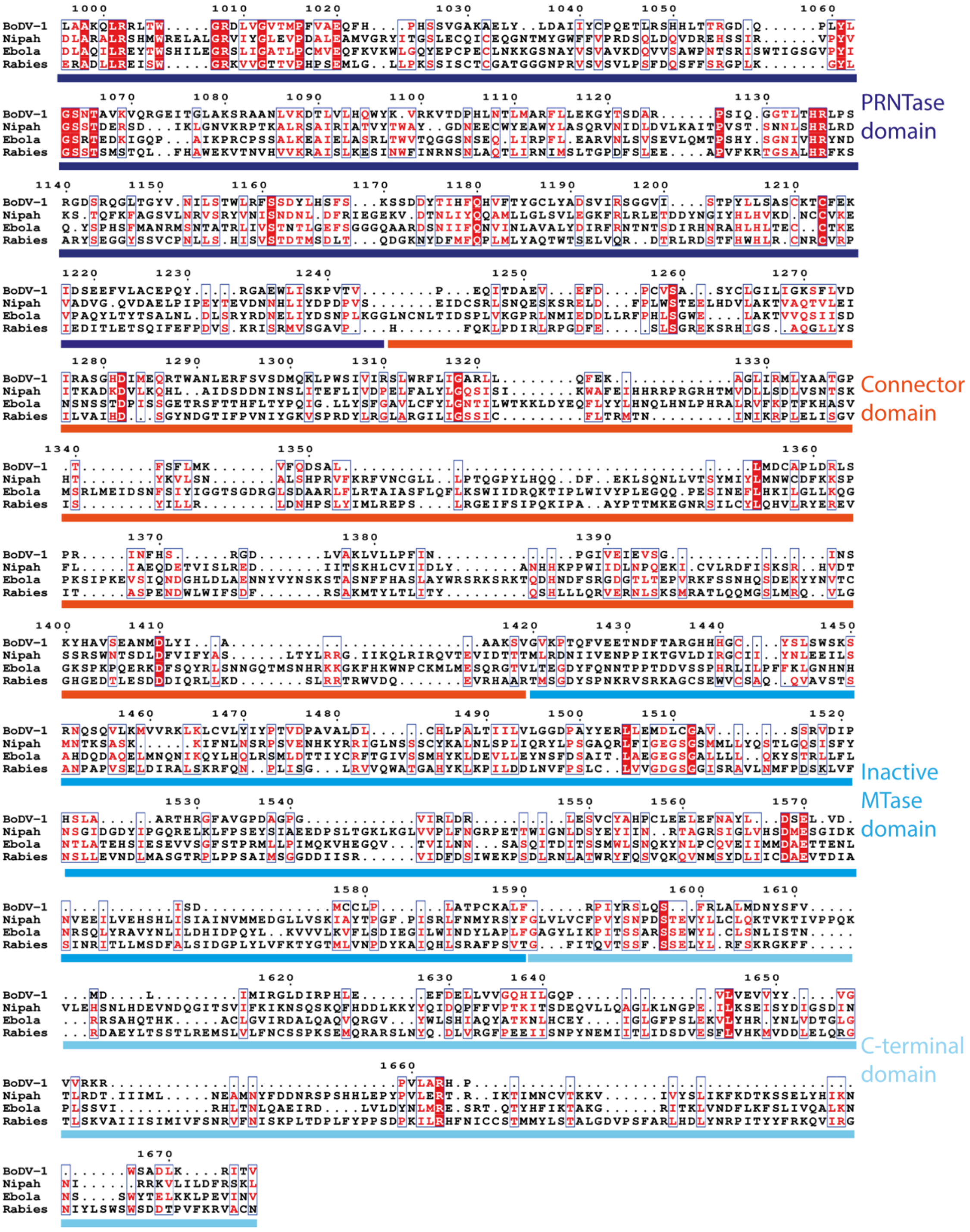

Supplementary figure 2. Sequence alignment of non-segmented *negative sense RNA virus* L-proteins. Sequence alignment for BoDV-1 (UniProt P0C799), Nipah virus (UniProt Q997F0), Ebola virus (UniProt Q8JPX5), and Rabies virus (UniProt Q8B6J5).

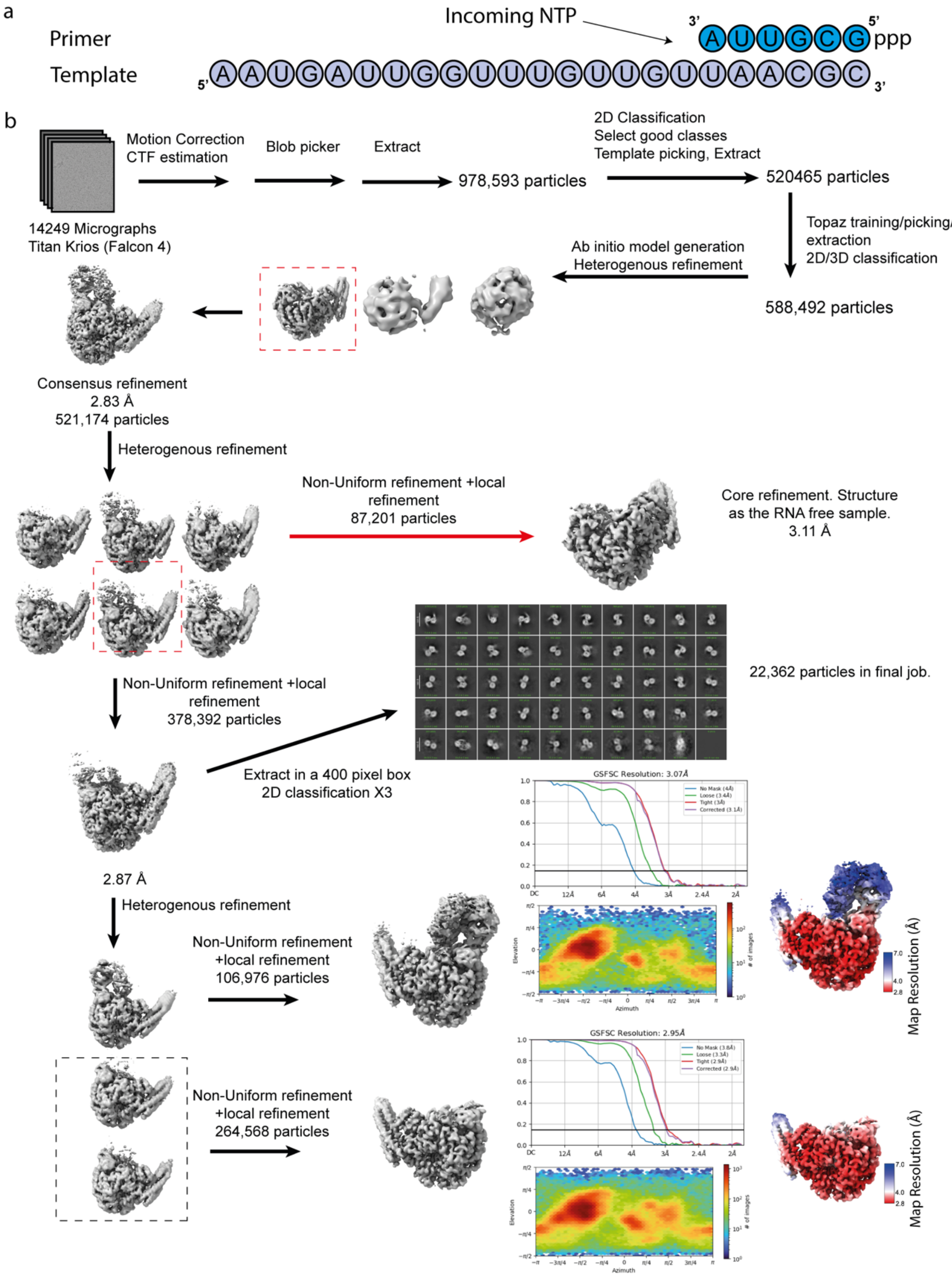

Supplementary figure 3. BoDV-1 sample 2 dataset CryoEM processing workflow. a) design and putative base pairing between the template and primer. b) cryoEM processing workflow.

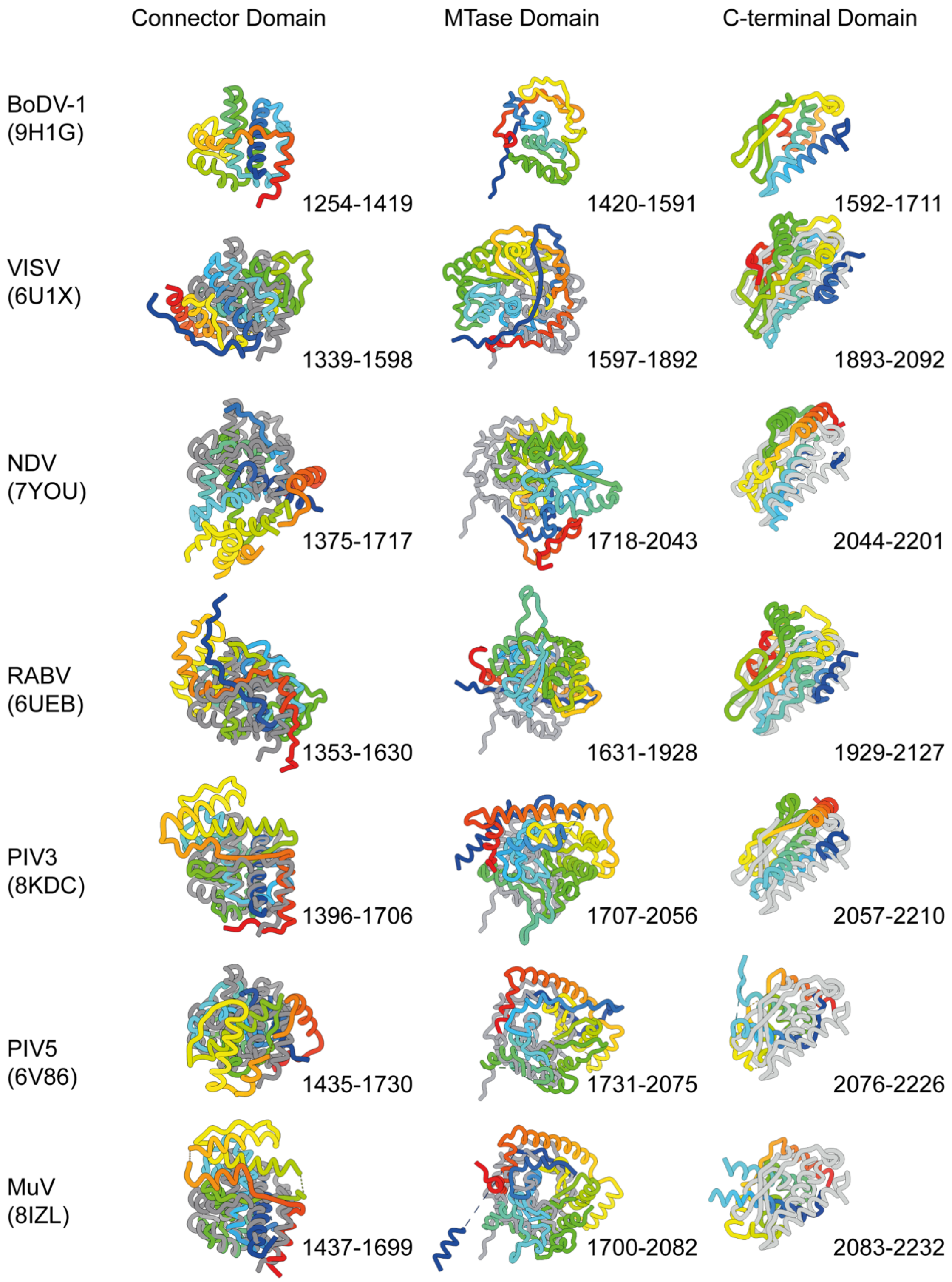

Supplementary figure 4. Comparison of the C-terminal regions from a selection of nsNSV L-proteins. BoDV-1 domains are shown for comparison in grey and are conserved between all panels. Domains from other nsNSV polymerase are coloured N-to-C termini in rainbow colouring (blue to red). Values next to the images are the residue range for the nsNSV domain.

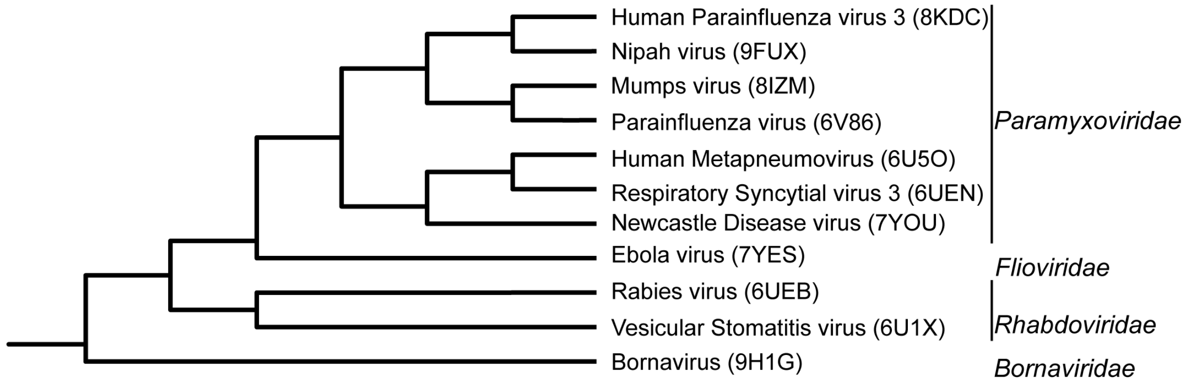

Supplementary figure 5. Structural phylogenetic analysis of experimentally determined *Mononegavirales* L protein models. PDB codes are shown in brackets.

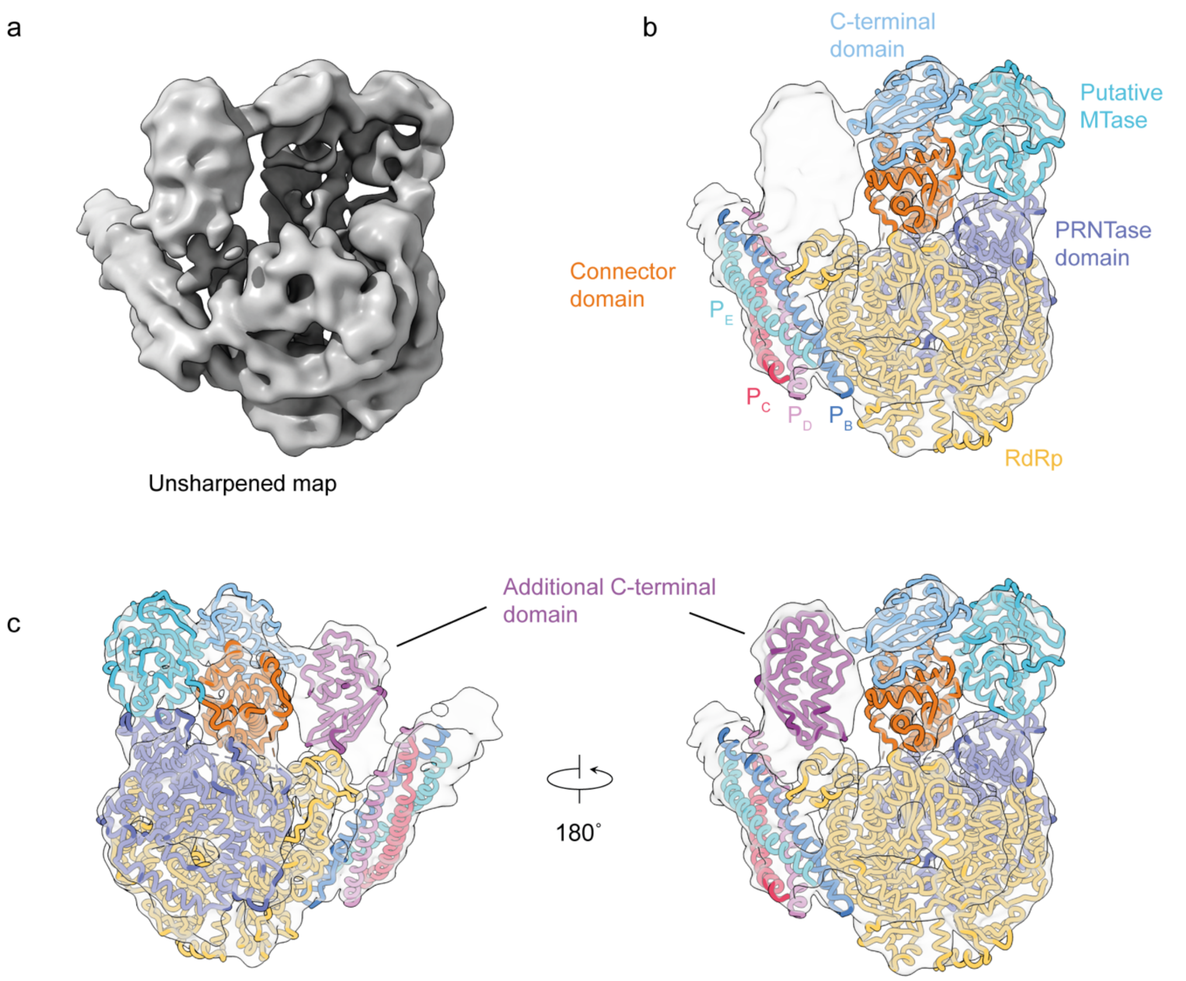

Supplementary figure 6. Observation of an additional copy of a CTD. a) Reconstruction and b) model showing the extra map density. c) potential fit of the copy of the CTD (purple). The fit shown is for illustrative size and shape of the density, but it cannot be positioned unambiguously given the quality of electron density.
